# Revisiting the use and effectiveness of patient-held records in rural Malawi

**DOI:** 10.1186/s12913-025-12844-0

**Published:** 2025-06-03

**Authors:** Amelia Taylor, Paul Kazembe

**Affiliations:** 1 Malawi University of Business and Applied Sciences, Blantyre, Malawi; 2 Chichewa Language Specialist, Machinga, Malawi; 3 INSPIRE Network, Kuyesera AI Lab, Blantyre, Malawi

**Keywords:** Patient-held records, Health passports, Medical record keeping, Health data, Privacy, Literacy, Malawi

## Abstract

**Background:**

Health Passports (HPs) are paper-based, patient-held records used in Malawi to document key details about the health condition of a patient and the care provided during medical visits.

**Aim:**

This paper assessed their use and effectiveness within the health data ecosystem, and their potential impact on patient care.

**Setting:**

The study setting was health facilities under the District Health Office in the Zomba District, Malawi.

**Methods:**

We undertook a qualitative study to determine the practices for data recording used by health care professionals and the importance placed on HPs by patients and professionals. We conducted an in-depth Focus Group Discussion with healthcare practitioners. Pages from completed HPs were analysed to extract practices for recording case presentation, diagnosis, and medication.

**Results:**

HPs were perceived to be beneficial to healthcare professionals as a means of transmitting information and communicate to each other the patient record history and treatment. Patients saw HPs solely as means of accessing services rather than as sources of personal health information with intrinsic value to them. Practices in recording patient notes varied considerably and we found many instances of illegible handwriting, and the use of abbreviations and shorthand that could be interpreted differently by clinicians and were not understood by patients. Language and communication barriers, low patient literacy and a general lack of privacy during consultations also had a significant negative effect on the use and effectiveness of HPs.

**Conclusion:**

There are significant gaps between the intended use and effectiveness of HPs in a clinical setting and the actual use by healthcare professionals and patients. Efforts to make sure that HPs can effectively fulfil the primary purpose of medical records are long overdue.

**Contribution:**

This study contributes to an under-researched area for understanding the use and effectiveness of patient-held records in LMICs.

**Supplementary Information:**

The online version contains supplementary material available at 10.1186/s12913-025-12844-0.

## Background

For the past 25 years, health passports (HP) have been an integral part of the health information systems of Malawi. HPs are patient-held medical records in the form of paper-based booklets and are used to document the health condition of the patient and the care received during each doctor visit. Malawi’s Health Information Systems policy, last updated in 2015, mandates that patients must present their HPs at each visit and healthcare providers must use these to update key details about the patient care pertaining to case presentation, diagnosis, care plan, treatments, and follow-up plan [1].

HPs enable a decentralised and portable health information system, as they allow patient records to be accessible to any healthcare professional, regardless of the hospital or facility the patient visits. In the period soon after their introduction, it was noted that HPs were well accepted by communities. Their use was accompanied by high expectations regarding their potential to enhance the quality of healthcare delivery by facilitating [2]:

- *Correct diagnosis*: Complete medical history in the HP could help providers make accurate diagnoses and could encourage careful documentation.
- *Quick diagnosis*: HPs could speed up diagnosis because they act as centralised medical records and thus remove the need to gather information from multiple systems.
- *Time saving*: Providers could save time by using HPs for patient history.
- *Client self-assessment*: HPs could educate clients about their health, helping both literate and illiterate individuals monitor and manage care.
- *Preventing fake drug records*: Recording prescriptions in HPs would allow patients to spot discrepancies in prescribed drugs and quantity and help prevent drug misuse.
- *Cost-sharing culture*: Paying for HPs would encourage patient responsibility and the habit of paying for healthcare services.
- *Provider choice*: Clients could have the freedom to choose any provider, who will be able to access their complete medical history through the HP, leading to better continuity of care and higher satisfaction.

For these expectations to be fully realised, HPs must be used correctly and consistently by both healthcare professionals and patients. This requires that the data recorded within HPs are of high quality and accuracy. Use and effectiveness go hand in hand because what is important is not only the use (e.g., measured by the recording of data) but also the capacity of HPs to be clearly understood and appropriately acted upon by both healthcare providers and patients. Without reliable and interpretable records, the potential benefits in diagnosis, treatment continuity, and patient empowerment are substantially diminished.

Direct studies on the use and effectiveness of HPs in Malawi have primarily focused on the design of HPs for maternal and child health, particularly in addressing misalignments between HPs and clinical care guidelines such as those for the proper documentation of child growth measurements and vaccination schedules [3, 4]. Other studies looked at the indirect use and role of HPs for transitioning to and integrating with digital records, or for monitoring and evaluation of the quality of health services and for supporting diagnosis and treatment. Several such studies after 2000 are mentioned in Table 1.

**Table 1:** Studied on the use of HPs in Malawi in connection to other health systems

Use of HPs	Ref.	Author (Year)	Key insights
Transition to or integration with digital EHRs	[5]	Tough, A.G. (2018)	HPs remain useful during digitisation. EHRs tend to be less well-organised than paper-based records systems.
	[6]	Msiska et al. (2017)	Despite the introduction of EHRs in 2001, paper-based records continue to be in use. Clinicians prefer HPs over EHRs for reliability and completeness.
	[7]	Tweya et al. (2016)	HPs are used alongside EHRs for antiretroviral therapy (ART) management. After each drug-dispensing visit, summary labels are printed and placed in the HPs.
	[8]	Weston, W. et al. (2016)	HPs were used to verify electronic records for pneumonia in children.
	[9]	Gadabu, O. J. et al. (2014)	A lack of HPs or inefficient use of HPs constitute a challenge in moving to digital EHRs due to the loss of historical and patient data.
	[10]	Allain, T. et al. (2011)	Barcodes are attached to HPs and are scanned during visits to retrieve patient records in EHRs.
Monitoring and evaluation	[11]	Aron, M.B et al. (2024)	HPs were used to assess service quality. Facility registers and HP data were more complete than the District Health Information System (DHIS2).
	[12]	Mauluka C. et al. (2022)	HPs were used to assess service provision. Incomplete HPs create discrepancies with DHIS2 and facility records, affecting program planning.
	[13]	Malpass A. et al. (2020)	ANC (Antenatal) attendance notes from woman HPs were used to assess malaria experience and knowledge among pregnant women.
Improving service quality	[14]	Simbeye A. et al. (2024)	Although child HPs are used to record vaccination data, including malaria vaccine uptake, mothers and caregivers had limited knowledge about vaccines, particularly regarding the number of doses and dosing schedule.
	[3]	Mndala, L., et al. (2024)	The paper discusses the re-design of the woman HP to address misalignment with care guidelines. HPs lacked comprehensive educational content for pregnant women, and clear instructions for healthcare providers on standard care and condition management.
	[15]	Stones, W. (2018)	Entries in woman HPs at the time of delivery can help assess the quality of antenatal care; integration into DHIS2 is recommended.
	[16]	Dasgupta, A. et al. (2015)	Woman HPs are underused for family planning data; separate cards are proposed to address data gaps.
Supporting diagnosis and treatment	[4]	Aukrust C. et al., (2023)	Child HPs aid clinical communication but lack diagnostic tools like growth charts, which are critical to support early diagnosis.

The health information system landscape in Malawi has changed considerably since HPs were introduced in the early 2000s. The number of records that health professionals must complete for each patient has increased, and many rural facilities lack adequate resources to maintain facility-based records, especially for outpatients who rely on HPs for their healthcare data [17].

Notably, recent digital health initiatives focussed on introducing Electronic Health Records (EHRs) to capture patient data. But their onsite implementation has mainly been feasible in urban, private, or larger government facilities [6, 18]. In Malawi and in many other African countries, paper-based records continue to be used alongside electronic records [17, 19]. A hierarchical system of data collection and transcription facilitates the recording and transmission of data from rural clinics to district facilities and, ultimately, to the national level, where it feeds into electronic systems [18].

Poor record management can lead to inaccurate diagnoses, delays in treatment, inefficiencies, and data gaps that affect decision-making. Paper-based records are frequently used to check the validity of data captured in electronic systems [20]. If a decline exists in the record-keeping practices in Malawian healthcare facilities, this will not only impact the quality of patient care but will also hinder healthcare professionals from transitioning to and upskilling in electronic record systems. Weak paper-based records also make digitisation more challenging. As early as 2014, it was noted that a lack of HPs, due to loss or damage, or inefficient use by health professionals constituted a challenge in moving to digital electronic health records (EHRs) due to loss of historical patient data [9].

Our investigation into the use and effectiveness of HPs is based on a study we conducted on language barriers in communication between health practitioners and patients in healthcare facilities in Malawi [21]. HPs were mentioned as a means of communicating with patients, but many patients did not understand or were confused by what was written in their notes. Part of the problem stemmed from the use of English and low patient literacy; however, these factors alone did not explain why healthcare practitioners failed to utilise patient history recorded in HPs when making diagnoses. These findings motivated us to revisit the utilisation and effectiveness use of HPs, and the importance placed on them by patients and health professionals.

This study engaged with healthcare professionals in rural clinics to assess the effectiveness and use of HPs for practitioners, patients, and the healthcare system. It aimed to identify key barriers that hindered the effective use of HPs by focussing on the following dimensions:

1. Usefulness and understanding:
  - The *quality of note making* in HPs by healthcare professionals.
  - The *perceived value* of HPs by both professionals and patients and their motivation to use and engage with the tool consistently.
  - The *effectiveness of HPs in facilitating communication* between professionals and patients necessary for understanding diagnosis and treatment.
2. Key factors that may affect use and understanding:
  - *Patient literacy* may impact patients’ ability to effectively communicate critical health information to healthcare practitioners, as well as their capacity to engage with and derive benefit from the content documented in their HPs.
  - *A lack of privacy* during medical consultations may hinder patient participation in health-related discussions and discourage the use of HPs.
  - *Language barriers* may impede the accurate documentation, interpretation, and effective utilisation of information contained in HPs.

### A brief history of HPs in Africa

HPs have been commonly used in Africa to record and track maternal and child health. Many of these passports align with WHO guidelines for home-based maternal records [22]. These HPs vary in format and complexity, ranging from simple antenatal notes or vaccination cards to more comprehensive booklets. They have been known under many names such as “maternal and child health booklets” in Kenya [23], “home-based maternal records” in Zimbabwe [24], “women-held notes”, “ante-natal cards” in Zambia [25], “child health cards” in Uganda [26], “road to health cards” in South Africa [27].

Booklets for general health are also used in some countries. A systematic review of literature reported in 2021 on the use of general HPs in three African countries: Lesotho, South Africa and Zambia [28]. Lesotho and Zambia have used general HPs for the longest time [29, 30]. Our literature review uncovered a few more publications from other African countries since 2021 (Table 2). In Zimbabwe, general HPs, called Patient Health Medical Records, do not have a standardised structure, patients being free to use any other affordable paper booklets of varying formats, including simple sheets of paper. Zimbabwe general HPs are legally recognised and admissible in court if authenticated by proper dating, official stamps, and signatures from medical practitioners. A recent study in Mozambique investigated the introduction of HPs for patients with cardiovascular diseases and advocated for the replacement of paper based records held at facilities with patient held records [31].

**Table 2:** Studies focussed on HPs in Africa after 2020 other than those used for mother and child HPs

Country	Type	Paper	Main points
Zimbabwe	P	Madziwa, P. K. et al. [32]	HPs lack a standardised structure, and patients opt for affordable booklets of varying formats. HPs are legally admissible if properly stamped and signed by facilities. Challenges in using HPs include unlawful manipulation, incomplete documentation, and loss by patients.
Africa	E	Towett, G. et al. [33]	A proposal for a multi-disease digital HP to support the management of non-communicable diseases (NCDs) and infectious diseases is made to improve data sharing and access across borders.
Lesotho, South Africa, Zambia	P	Joseph, L. et al. [28]	The systematic review covers general HPs used in 3 African countries and finds that their use by healthcare providers is suboptimal due to limited protocols and training. There is little recording of health outcomes, and the utility of HPs for patients is unclear.
Mozambique	P	Lumbandali, N. et al. [31]	The study found support for the replacement of paper-based records held at facilities with patient-held medical records for the management of cardiovascular diseases.

*P* paper-based and *E* electronic HPs

In many countries, patient-held records are copies of or extracts from facility-kept records, their primary purpose being to inform patients and encourage participation in the care plan. Factors related to the suitability of information captured in HPs for different age groups, differences in the patient and doctor understanding of the function of HPs, and confusion over records ownership have been shown to negatively affect adoption [34]. In African countries, studies tend to emphasise the importance and the positive impact of HPs on patient care [28]. This is primarily due to the fact that handwritten HPs were introduced to help address the challenges arising from a lack of integrated health information systems capable of facilitating data sharing across healthcare facilities. HPs were conceptualised as portable health records that patients carry and present during visits to any healthcare provider or facility. Recently suggestions have been made for the use of electronic HPs, particularly for the management of non-communicable and infectious diseases [33]. However, there are very few studies on the effectiveness of general HPs other than those for maternal and child health, making their impact on health outcomes uncertain [28].

## Methods

### Design

We conducted a qualitative study on the use and effectiveness of the general HPs in Malawi using a Focus Group Discussion (FGD) and document analysis of sample pages from HPs. Participants for the FGD were selected purposively. The researchers engaged previously with the participants in a study on language use in health facilities in Zomba District where some issues pertaining to HPs were mentioned [21]. Participants were knowledgeable about the subject matter and/or held leadership positions in their respective facilities. Participants directly interfaced with patients and utilised the HPs to record clinical encounters. They expressed interest to participate in an in-depth discussion about HPs and language issues. Participants were contacted by telephone, followed by an official invitation and relevant documents sent via email.

A total of 10 participants were included in the FGD: 3 were women and 7 were men. There were 6 clinicians, two nurses/midwifes, one data officer, and one laboratory technician. Participants working experience ranged from 5 years (2 participants) to over 20 years (3 participants). This helped us cover the use of HPs from varying perspectives and exposures. At the time of the study the practitioners were based at 9 different clinics and had experience of working in more than one clinic, both government and private. The clinics represented were: Chipini, H. Paker, Machinga, Mmambo, Naisi, Matawale, Machinjiri, Bimbi, and Chanco. H. Parker, although a government clinic was run by a non-profit organisation and offered some paid services such as laboratory tests. Chanco clinic was attached to the University of Malawi but served also non-university staff. The rest were solely government run. The figure in Appendix 1 shows the geographical area, facilities covered and their catchment areas mostly being very rural areas.

No participant refused to participate or dropped out. The FGD took place in September 2024 in a conference room at a lodge in the centre of Zomba city to ensure easy access for all participants. In addition to the two researchers and participants, a note taker was present during the focus group discussion (FGD). This note taker was known to the research team and acted in this capacity in other studies.

A FGD guide was provided to all participants at the beginning of the FGD. While no formal repeat interviews were carried out, we stayed in touch with participants and followed up with some questions clarifying items by WhatsApp or phone. The sessions were audio recorded. The FGD consisted of two parts: a morning session lasting approximately 2.5 h and an afternoon session of about 2 h. Transcripts and summary of key findings were returned to participants by email for their comment and/or correction. The FGD was conducted in English. Translation was rarely needed during the transcription for Chichewa expressions that participants used to contextualise their answers. Such occurrences were few and always accompanied by their explanation in English. The research team had expertise in Chichewa and linguistic research. The second author is a secondary school teacher with experience of over 15 years of teaching Chichewa as a first language. The first author has lived and worked in Malawi for the last 12 years, has basic competency in Chichewa and has encountered language issues and challenges in higher education in Malawi. The first author conducted research in language issues emerging during data collection for COVID-19 and has developed datasets for Chichewa text for machine translation. 

We used document analysis to assess the quality of the medical notes recorded in HPs by professionals. The data we analysed in this study comprised of:

- Sixty-one unique sample pages of completed pages in several health passports. These were obtained from patients who participated in the first study [21]. These were analysed to assess health professionals’ practices in documenting patient notes, as well as the quality of the coding applied.
- Transcriptions of audio recordings and the notes taken during the FGD were analysed for key research themes.
- Key health policy and guideline documents were consulted to establish any mention of the use of HPs, and the existence of guidelines and procedures for recording patient notes.

Two data coders were involved in data analysis. The themes were identified in advance and were developed by the authors and were informed by the findings of the language study [21]. The main themes and lines of inquiry are presented in Table 3. The complete FGD guide is presented in the supplementary material and together with the consent form are available on the Open Science Foundation [35].

**Table 3:** Study questions and themes of inquiry. The themes 1–7 were used for the FDG and 7–9 for document analysis

#	Themes	Questions considered
1.	Provider use	How do healthcare professionals use the information in the HP? Have you done any training on how to record notes, or how to make entries in the HPs? Are there symbols that are commonly used?
2.	Importance	What value do you think healthcare professionals place on HPs? What value do you think patients place on HPs?
3.	Effectiveness	Do patients ask for clarifications about what is written on their HPS? Do you discuss with patients what is written on their HPs during consultation? How could HPs become more useful to healthcare professionals and patients?
4.	Impact of patient literacy	What do you think is the impact of low literacy on the quality of information exchanged during consultations? In what way do you think low literacy affect the ability of patients to express their symptoms and conditions? In what way do you think literacy could or does affect the ability of patients to express to understand their diagnosis and treatment?
5.	Language and communication challenges	Do you think there is a case for writing symptoms and diagnoses in Chichewa or the language of the patient in the HP? Does the use of HPs improve the communication between practitioners and patients?
6.	Issues of privacy	To what extent do you think a lack of privacy affects good communication? In what way do you think a lack of privacy affects the quality of healthcare? What kind of privacy situations occur? What can be done?
7.	History	When were HPs introduced in Malawi? What was the intention for their introduction?
8.	Link to eHealth systems	How are HPs used in connection with registers, tally sheets at facilities? How are HPs used in relation to other electronic records? How does their use impact training and readiness for full digital health systems?
9.	Other issues	Who owns the patients’ health records?

**Fig. 1:**
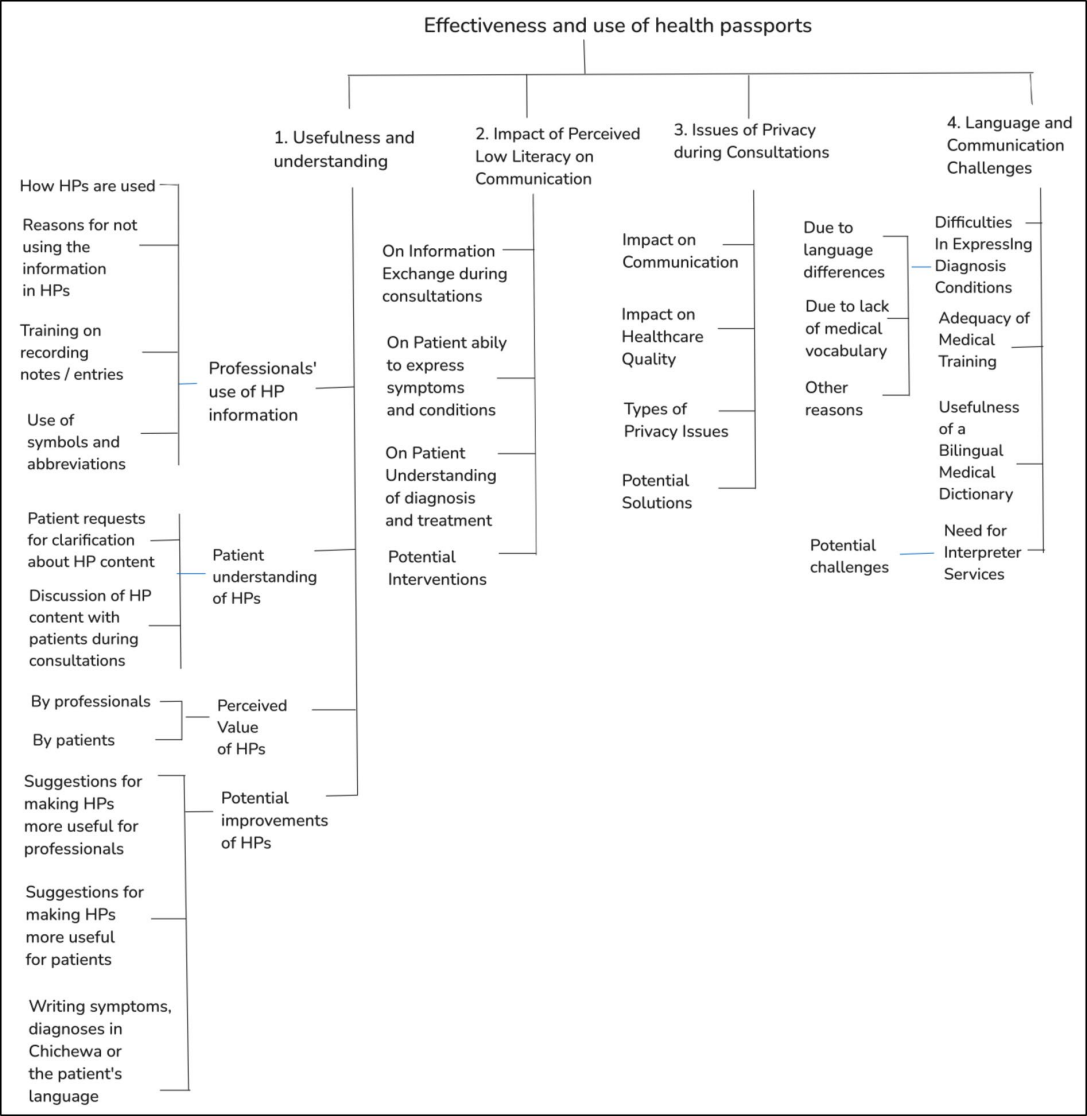
Coding tree use in quantitative analysis of the FGD transcripts

The coding tree is given in Fig. 1. Data analysis was done by highlighting words and phrases in Word and using Excel to record and summarise the codes. Images from HPs were manually reviewed and we extracted abbreviations and signs used in tabular format in Excel.

Data saturation was discussed among the research team and with the participants. Participants provided feedback on the summary of the findings. We ensured consistency between the data presented and the findings, and the major themes were clearly presented in the findings. Participant quotations were used to illustrate the themes and findings, although participant numbers were not included due to the small sample size.

### Research clearance and ethics

Participants were fully informed about the aims and methods of the research and were given relevant background materials about the study. They were assured of anonymity and confidentiality. All data were anonymised in the reporting process. Participants expressed that the research topic was of high relevance to their professional experience and had benefited from the discussions. Informed consent was obtained in writing from all subjects. The study posed no foreseeable risks and involved no interventions.

At the time when the research was conducted, the university where the researchers worked did not have an institutional ethics review board. Given the absence of an ethics board at the university at the time, this study followed ethical procedures that are used for similar research projects by ensuring local approvals and strict adherence to international ethical standards. Formal permission was obtained from the Zomba District Health Office to engage with both health professionals and patients for the purposes of exploring the use of language in written and verbal health communication.

We would like to give a detailed explanation of informed consent for samples from HPs. These were collected during a previous study. All patients who provided samples from their HPs gave their informed consent verbally. We took the view that emphasising signed consent forms risks shifting the focus toward procedural compliance rather than fostering genuine understanding and voluntary participation. Hence, we adopted an approach that encouraged free and informed participation. In rural Malawian contexts, seeking permission through community leaders, in this case from facilities, is often viewed by potential participants as an important and respectful step in the research process.

This approach aligns with findings from a very recent study conducted in Zambia, a country with cultural similarities to Malawi which highlighted that written consent may not always reflect culturally appropriate expressions of agreement [36]. The formal nature of written consent can feel unnatural and may hinder rapport between researchers and participants. The requirement to sign documents may generate mistrust, anxiety, or stress, particularly in Malawi where formal documentation is associated with authority.

The following was the setting: upon obtaining permission from the head of a facility, the authors sat in a designated area and patients were informed of our presence by the pharmacist or the clinician after their consultation. Patients who were willing to participate would then approach us and obtained further information about the study. They were all asked for their consent before proceeding to completing a questionnaire on language use during consultations. Additional informed verbal consent was obtained from patients who wanted to contribute with pages from their health passport. The purpose was clearly stated: to help us understand the quality of the notes, use of language and the use of medical terminology.

## Results

### History of the use of HPs

HPs were introduced nationally during early 2000 s by the Fourth National Health Plan 1999–2004 [37]. The use of HPs is clearly stated by the National Health Information System Policy (NHIS) [1]. Table 4 contains the paragraphs from the NHIS that mention the use of HPs. Health practitioners are required to update the HPs during each visit with essential medical information from patient records, which are expected to be maintained and kept at facilities. The policy also specifies the type of information that should be recorded in HPs: proposed diagnosis, plan of care, treatment given and follow-up date. The NHIS policy has not been updated since 2015, nor has it been supplemented with practical guidelines on the documentation of health records.

**Table 4:** Data ownership and use of health passports as per the Malawi National health Information System Policy 2015

14. Data Ownership 14.1 The ownership of any health-related data shall rest with the Ministry of Health. 14.2 Each patient or any person shall have a personal health passport which will contain their key personal health details which shall freely be taken home by the patient for ease of reference and personal monitoring of their health matters, in addition to the individual patient records kept in the facility. 14.3 All health facilities and health practitioners whether in public or private facilities shall ensure that patients/persons seeking care in their facilities have, in their possession personal health passports in addition to patient files kept in the facility. 14.4 All health facilities and health practitioners whether in public or private facilities shall ensure that patients/persons seeking medical care present health passports to their care providers. 14.5 All Health Practitioners be it in public or private facilities shall update their patients’ health passports with relevant key details extracted from patient files kept at the facility each time they are presented to a health practitioner at the time of seeking care. 14.6 Updates of the health passport in the custody of the patient shall include proposed diagnosis, plan of care, treatment given and follow-up date. 14.7 All sharing of patient personal identifiable information with third parties by public health facilities, private health facilities, insurance companies or individual health practitioners shall be done only with written consent from the patient or their caregivers where the patient is a minor.	

HPs are used as sources of data for other facility registers and are well integrated in the processes of rural clinics. The following procedure for data collection was observed in our first study and confirmed during the FGD: data clerks had a dedicated desk space and collected data from patients after they finished the medical consultation. The clerks transcribed data from HPs into OPD (Out-patients department) and other registers according to the services that the client received. Generally, data from facility registers were compiled, aggregated and reported monthly using both programme-specific reports (e.g. maternity, HIV programs) and composite reports. Data collection and aggregation was the responsibility of Health Management Information Systems officers who supervised the work of the data clerks. Malawi uses DHIS1, an electronic system for aggregating routine facility and community service data [18]. HPs are routinely used to document critical information for patient referrals to central hospitals, and they also play a role in reconstructing medical histories, such as identifying conditions or symptoms relevant to determining causes of death, often in conjunction with other records. After the death of a patient, their HP should be returned by relatives to the closest health facility.

#### The format of HPs has remained largely unchanged since their introduction

**Fig. 2:**
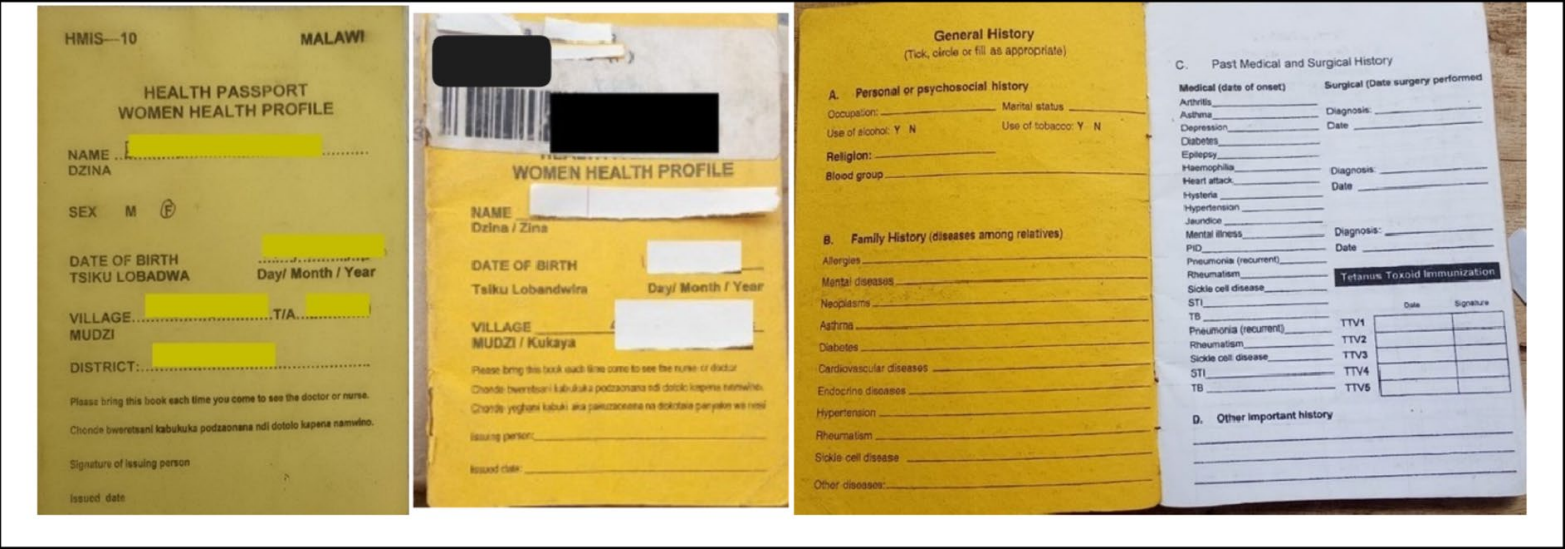
Samples HPs for women (yellow cover), cover and inside cover and front sheet

**Fig. 3:**
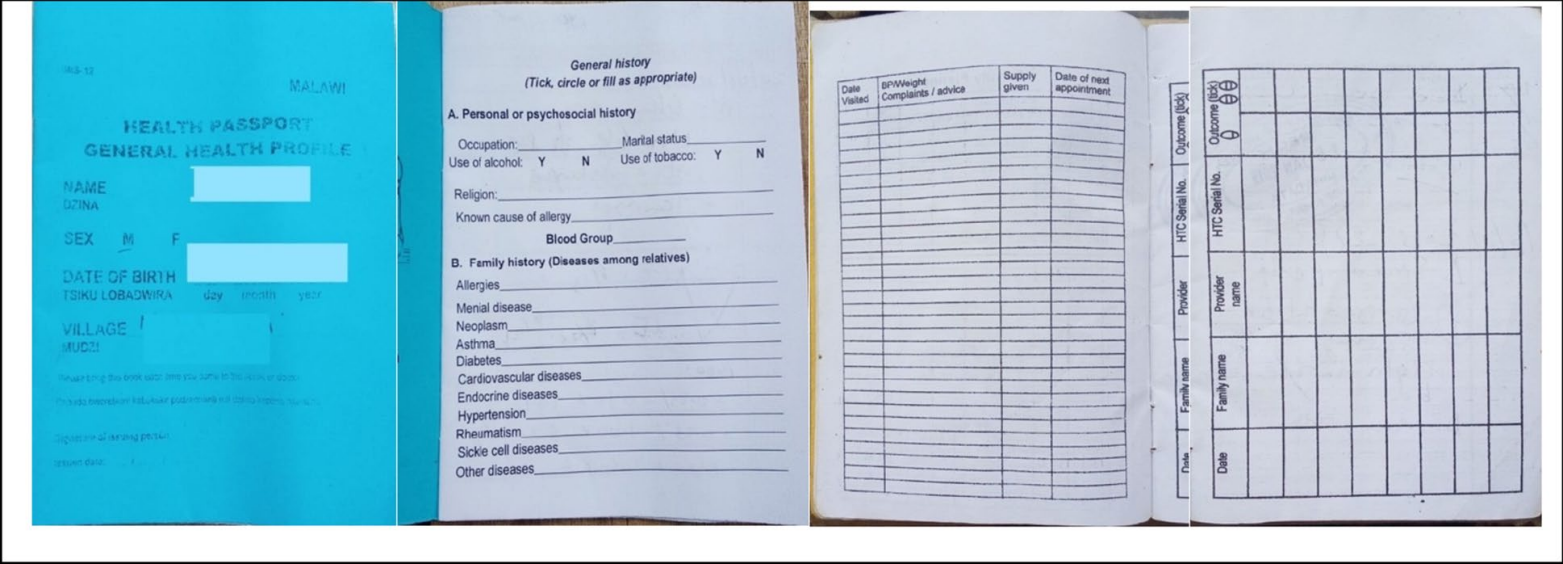
Samples of HPs for men: general history page, list of visits, HIV Testing and Counselling sessions

The official HPs format and colour scheme has remained largely unchanged over time since their introduction in early 2000s. HPs are colour coded. The women HPs have a yellow cover and contain standardised sections for immunisation, family planning, previous childbirth history (Fig. 2). A girl is given a women’s health booklet when she reaches puberty. For small girls, the HPs are pink, and for small boys, they are blue. The child HPs are issued at birth, and contain standardised sections for vitamin A, immunisation and growth monitoring. HPs for male adults are blue in colour. The men HPs are usually annexed to the boy child health booklet for continuous recording of assessment and care (Fig. 3).

The cover page of the HP includes the patient’s name, date of birth, village, traditional authority (T/A), and district, these details can be filled in either by the patient or by the healthcare professional (Figs. 2 and 3).

Currently the format and quality of the booklets are not controlled. Patients can buy them from vendors outsides clinics. Participants recalled a time when HPs were issued only by recognised organisations and had a recommended number of pages and quality. Presently many HPs have very few pages limiting their use for multiple visits. HPs sell at K800, and, although this seems small, their cost has become a burden and a deterrent for many poor patients.

### Usefulness and understanding

#### How do doctors and healthcare providers make entries in HPs?

Notes in HPs were written by hand during the consultation. All notes are in English. All notes were dated. Some had signatures and some had stamps. Many contained illegible handwriting. We showed several sample pages from HPs to participants during the FGD and most of them agreed that the legibility of the writing was inadequate.

#### Limited space and high workload

HPs have a limited number of pages, something that was suggested as a cause for brief and sometimes incomplete notes. Healthcare professionals handled a large number of patients, and the time available for consultations was too short to allow providers to write detailed explanations about diagnoses and conditions.

#### Professionals’ use of HPs

Participants used the HPs regularly, but there were exceptions. In private clinics and for clients who utilised private health insurance, HPs were not used. Instead, patients were given printed or handwritten notes with test results and medication prescriptions, often tied to billing. Insurance schemes had dedicated forms for registering and recording patient visits and documenting care. This omission in the use of HPs was considered negligent, as it contributed to gaps in medical history and undermined the HP’s role as a comprehensive health record.

#### Insufficient patient medical information was recorded

**Fig. 4:**
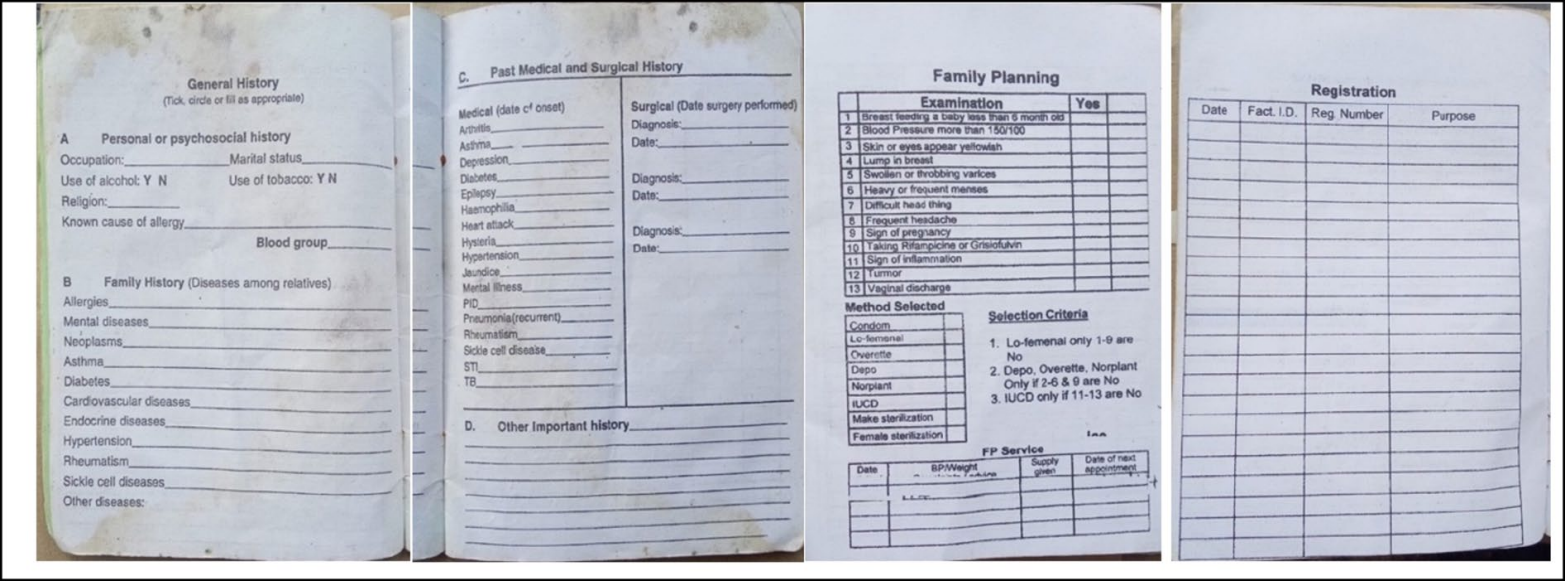
General history pages, family planning page and registration in HPs. The registration page records visits made to any health facility

A general section divided into three parts is found on the first pages of HPs and contains the summary of the medical history of the client. Part A is for the psychosocial history and includes occupation, marital status, alcohol and tobacco use, religion, known allergies, and blood group. Part B contains family history, including known allergies, mental health conditions, asthma, and other relevant conditions. Part C is dedicated to past medical and surgical history. We found that the general history section was blank for most patients (Fig. 4). X-rays and other test results were not routinely provided to patients or when they are available, they are not attached to the HP.

#### Use of symbols and shortcuts

We found the presence of many abbreviations and symbols. Opinions on the use of abbreviations were divided. Some believed that all words should be written in full to ensure that the patient and other professionals can easily understand the information written in HPs. Some referred to the practice of using disease codes in OPD registers and deduced that this practice also applied to notes made in HPs.

The use of shorthand and abbreviation was also seen as a mark of experience because they were frequently used by consultants; junior staff tended to write longer notes using complete words.

*"If you meet a consultant, they advise you to write clearly in the health passport. They write in professional language which nurses giving the medication will understand."*

*“This (the use of abbreviations) was something that was learnt in school and is acceptable by law.”*

“In the OPR, information is written in short form which is acceptable by government.”

*"The Malawi Standards for Treatments Guidelines accepts the short forms…one needs to refresh one’s memory."*

When asked, participants could not point to any laws, guidelines or training that specifically contained instructions on the use of abbreviations apart from the Malawi Standards for Treatments Guidelines (MSTG) which contained symbols for medication and dosages.

An incident was recounted in which a patient insisted that his notes were written in full in his HP and requested that the health professional rewrite them to ensure no shortcuts were used.

Participants remarked that the use of long expressions and full description of diagnosis cannot be perfectly achieved due to the current design and length of the HPs and due to time constraints. Hence, they argued that the use of shortcuts was acceptable. One participant disagreed and was of the view that long queues or a lack of page space should not prevent health professionals from making fully understandable entries in the health passport.

*“And because of the long queues experienced in clinics, health centers and hospitals ‘we make a lot of short cuts’ (use of signs/short medical expressions) aiming at serving many by just ensuring that the long queues are all deflated and served regardless of the quality of service offered and we usually provide a not fully articulated information in the health passbook*. *Though it is an important information packed book, the spaces in it are so limited ‘its brief, we can’t write everything’ only to accommodate short forms of writings in making entries.”*

Table 5 is an example of notes transcribed from a patient HP: symptoms were written mainly in full words in a section denoted by “Cl” probably standing for “Clinical”. The original image was not shown due to the possibility that handwriting or other information may be used to identify the patient of the healthcare professional who wrote them. The treatment appears in a section denoted by the letter “P” (for plan) and there were codes and symbols for drugs and dosages. The date of the visit was recorded on the top left of the page.

**Table 5:**
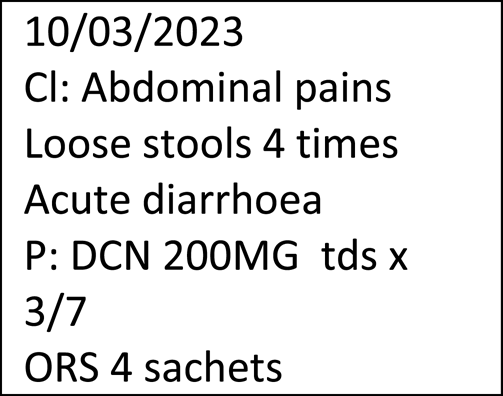
Example of clinician notes in a health passport transcribed from the original handwriting

#### Symbols used

Symbols used in the HPs were extracted and fell into seven categories: charting (7 types of symbols) and drug-related (21 symbols) were most common, followed by diagnosis or condition (19 symbols), laboratory (18 symbols), pharmacy (17 symbols), vital signs (17 symbols) and anatomy (1 symbol) abbreviations.

Table 6 shows how many different symbols were counted for each category. In Table 7 in Appendix 2 we list all the abbreviations found in the sample HPs alongside their most likely meaning as obtained via discussions with health practitioners. For example, there were 12 different symbols used for charting, e.g. *Hx* (History of) and *C/O* (Chief Complaint). Charting abbreviations referred to taking down the history of a case but were also used to demarcate sections such as those dedicated to treatment plans and diagnosis. Not all abbreviations were used consistently: for example, some referred to treatment plan using the word *Plan*, others used the letter *P* and others used the more generally accepted *Rx*.

**Table 6:** Types of abbreviations used

Type	No. Abbreviations
Charting	13
Drug	11
Diagnosis/Condition	8
Laboratory	7
Pharmacy	5
Vital Signs	5
Anatomy	1
Total	50

Diagnoses were surprisingly few: sepsis, malaria, RTIs, and skin conditions appeared most frequently. Some were written in full, such as sepsis, while others, particularly respiratory conditions, were often abbreviated, e.g. ARI for Acute Respiratory Infection. Reference to social history such as smoking habits, or to occupation were very few: one such note referred to voice loss suffered by a teacher. Family history and past medical history were not included.

The notes did not contain details of the physical examination of patients or history of illnesses. Reference to anatomy was made in a summary way with few descriptions. There was little to no reference to procedures such as CT scans or X-rays. This could be because most clinics did not have access to such facilities. Most tests mentioned were rapid tests except for a few which may have been recorded by private clinics with functioning laboratories.

There was a higher uniformity in the recording of antibiotic drugs. Names of common drugs and their abbreviations appeared to be well known to healthcare professionals, e.g. Amoxyl and CTX. Some drugs appear in their full name such as Artesunate, being is the first-line treatment for children or adults with severe malaria.

#### Use of signature and stamps

We observed various ways in which practitioners signed their notes in HPs: some used only their signature, while others used their initials or a combination of their initials and surname. All were in lowercase letters. None used capital letters, a standard that is considered good practice in many medical settings. Some notes did not have any signature at all. However, our findings could be limited by the fact that we only captured a sample of pages sometimes one from each passport.

We learnt that private clinics and those run by the Christian Health Association of Malawi, a private organisation, used stamps. Stamps were used for verification to confirm that a patient has gone through data capturing before receiving treatment at the pharmacy. The stamp was used when referring patients to a central hospital, as part of the protocol for accessing tertiary care. The referral procedures required that patients must first be seen by their local facility who can then refer them a central hospital.

#### There was a lack of guidelines and policies on medical notes recording

Participants did not receive any specific training on the use of HPs. One participant referred to consulting international books on good practice regarding communication with patients, but no national or specific guidelines for recording patient notes in HPs exist in Malawi. Some participants felt that the general note-taking skills learned in school should be sufficient. The Malawi Standards for Treatment Guidelines (MSTG) contained guidelines for prescribing medication and included a list of common medical abbreviations for drugs and conditions, and guidelines for in-patient prescriptions [38]. We noted that many antibiotic treatment plans followed the MSTG guidelines. In contrast to registers, where a defined list of abbreviations for medical conditions was available and consistently used, the diagnoses recorded in HPs were not standardised.

#### Discussion and use of HP content with patients during consultations

The HPs were initially envisaged to support the process of shared decision making by which practitioners and patients make informed decision on diagnostic and treatment. HPs should put reliable information in the patients’ hand, and this information could open discussions and information sharing between patients and practitioners during visits. We found that due to language barriers and low literacy, this was hard to achieve. Patients understood little of what was written in their health passports. Typically, the information prioritised by both professionals and patients was the prescribed treatment plan.

“Lay patients i.e. villagers and community members including some health workers cannot understand some or all medical entries made in the health books. The illiterates remain the main victim of this because majority of them never understand what is written in their health books. Patients will only just see the medicine given such as PCM (paracetamol), Panadol, metronidazole etc., but in majority do not know how the doctor’s conclusive diagnosis was drawn from the symptoms explained. They don’t know what they are suffering from.”

Healthcare professionals tended to prioritise communicating drug instructions and treatment schedules rather than explaining medical conditions to their patients, an approach that was observed in patients’ own focus on accessing treatment rather than understanding the diagnosis.

While health professionals generally viewed HPs as more useful to them than to their patients, they did not habitually refer to past entries in the HPs. They tended to rely primarily on the patient presentation during the visit. The patient’s account was considered more important than explanations of symptoms provided by a guardian, as it was believed that such explanations could be prone to exaggeration.

The perception and attitude of some professionals was also regarded as a barrier to engagement with the HPs. Some professionals wanted to have control over consultations and resisted being guided by the information in the HP. Power dynamics came into play as well and some practitioners dismissed patients who handed over their HPs unopened, telling them to “bring it already opened to the right empty page.” While the intention behind this request might have been to save time, it could have resulted in practitioners unintentionally signalling to patients that their medical history was not important.

#### Patients requests for clarifications about HP content

Majority of patients did not ask for clarification on the entries that a doctor made in the health pass books, due to:

- poor rapport between a patient and the doctor.
- the doctors appeared to do things in a hurry when serving long queues of patients.
- low level of literacy among majority of the patients.
- loss of confidence and low self -esteem among many patients who in majority were illiterate.

Some discussions about medication administration took place with the pharmacist, who gave instructions based on the doctor’s prescription. Some patients expressed preferences for certain medications, often due to past experiences. This was especially common in private clinics. For example, a patient who felt symptoms of malaria might insist on being prescribed anti-malaria medication, even if the lab results were negative.

“Sometimes when a medication is out of stock, the patients might be given (by the pharmacist) a different drug than the one prescribed in the HP.”

Some patients might refuse to comply with taking the dispensed drug when this differed from the one prescribed, a situation that was often caused by unavailability of certain drugs.

#### Special needs patients

Communication between healthcare providers and patients with special needs was noted as a significant challenge during consultations. Sign language skills, such as gestures, were partially covered during medical training, but not adequately enough to equip professionals to communicate effectively with special needs patients.

#### Perceived value of HPs

All professionals agreed that HPs were valuable to their work and are an important tool for recording and transmitting information to colleagues and patients. They thought that patients viewed the HPs primarily as a requirement for accessing healthcare, rather than valuing the information these contained. Women and the elderly were more likely to bring their HPs to medical visits. Cases of damaged, lost, or misplaced HPs were common. Patients had limited awareness of the importance of maintaining a single, reliable health record.

Many professionals admitted feeling guilty about not educating patients on the importance of the HP. Participants identified several common problems and situations:

- some patients did not have a HP, the majority did not afford to buy it at K800.
- some patients had several HPs. Those on Antiretroviral therapy (ART) for treating HIV infections had separate confidential HPs, which along with other treatment cards were sometimes kept at ART clinics. Notably, the ART HPs were only presented to ART health officers and never to other clinicians.
- some patients intentionally left their HPs at home to avoid discussions about treatment or underlying conditions, especially if patients failed to adhere to the prescribed care.
- some patients accepted their HPs to be used by other relatives.
- all entries were done in English and used medical terms that could not be well understood by most patients.

#### Affordability and access issues

Concern about costs were frequently mentioned, some patients resorted to borrowing HPs from others, and there were cases in which a man might use his wife’s HP. The NHIS policy does not explicitly state that care will be denied if a patient arrives at the facility without their HP, but places responsibility on health facilities to ensure that patients present their HP during visits. Some professionals said that they refused to see patients that did not present their HPs for consultation.

#### Potential improvements of HPs

Potential improvements were identified during discussions. However, it was felt that further, more focused follow-up studies are needed in order to better understand what can be most effectively implemented and where efforts should be prioritised. Below, we present the most clearly expressed recommendations:

- Standardise the format, quality and length of HPs.
- Address concerns around costs.
- Educate professionals and patients on the value and importance of HPs.
- Provide guidelines for note-making in HPs to improve the quality and effectiveness of the medical records.
- Work towards improving the rapport between professionals and patients during consultations to encourage sharing of information.

### Impact of perceived low literacy on communication

Low literacy was an important factor for the quality of the information patients received. A cultural preference for verbal communication over written notes by healthcare professionals was acknowledged. Professionals should provide good explanations to patients and ensure they understand the consequence of their actions in not following a treatment correctly, especially because some patients were afraid to ask follow-up questions. Many patients were not able to repeat the instructions given to them. Good rapport with the medical professional was seen to be very important. Most illiterate people had low self-esteem and feared when they met clinicians hence they struggled to express themselves for these reasons. Literate patients were given better attention and were received more favourably by healthcare professionals than were illiterate people.

“There is a problem in the way health professionals communicate with those who come for medical help, at the clinics. Healthy workers provide half -baked information and that after physical examinations, observations, medication and diagnosis, we don’t ask for feedback from the patients. The illiterates remain the main victim of this because majority of them never understand what is written in their health books.”

On the other hand, literate and educated people were difficult, they researched their conditions on the internet and were forceful in asking to receive specific medication. Patients who attended private hospitals sometimes sought care for trivial reasons (“they are not really sick”) and were insisting be given the medication they wanted.

### Issues of privacy during consultations

Both language and privacy significantly influenced the use and effectiveness of HPs. Inadequate privacy during consultations discouraged patients from sharing essential information about their symptoms or conditions and reduced their willingness to use HPs. Participants identified privacy as a key concern, often linked to poorly designed facilities that allowed others to overhear sensitive discussions. Additionally, insensitive attitudes from doctors during consultations further undermined patient trust and engagement.

### Language and communication challenges

Entries in all medical records, including in HPs, were done in English [21]. Participants considered that making entries in Chichewa would not solve all the problems of communication because not all patients were fluent in Chichewa or knew medical terms in Chichewa. This also assumed that health professionals learnt and understood medial terms in Chichewa, but many did not have the required vocabulary. Many medical terms in English did not have direct equivalent terms in local languages. Their translations were long descriptions and would take more space on paper.

Differences in dialects mean that some term have multiple meanings depending on the tribe, region or place. For instance, in Nkhotakhota among the Tonga tribe, *ntchofu* means hernia while in common Chichewa, *ntchofu* means stomach discomfort that results into nausea and vomiting.

However, practitioners considered entries in HPs should be explained verbally to the patients in their language and suggested that diagnoses could appear in both English and local language.

“It will be good if entries are put in local languages because the health worker will be able to elaborate the diagnosis to the patient and the patient will be able to understand, in local terms, the diagnosis, that is written in his/her health passport.”

Having access to interpreters was looked at favorably if interpretation services would be offered in all local languages. Preference for Chichewa might be construed as a political move to advance one language over others. The interpreter should be specifically trained to interpret medical conversations. Additionally, patients should be asked to consent to the use of an interpreter otherwise their presence could inhibit patients. It was suggested that applications of AI and technology could offer a better solution over using human interpreters:

*"A digital translator would be more preferable than a third party because that would be a good communication direct between clinicians and clients."*

*“A translator would ease the communication between health workers and clients”.(…) It will be more work for the health workers to learn the local languages, but this will benefit the client.”*

*"The moment you add a third party to the conversation, the patient may feel uncomfortable, a lot of Malawians value privacy."*

#### Participants did not support staff deployment based on Language competence

Deploying medical personnel based solely on their language proficiency was not seen as an ideal solution to language barriers. Some health professionals preferred not to work in their home areas where their mother tongue was spoken, fearing a lack of respect from those who knew them. Additionally, such deployment was seen as unintentionally reinforcing tribalism, stereotypes about literacy levels, and cultural beliefs. Others worried that working in their home village or town may lead to increased absenteeism or lateness, as personal obligations could take precedence over their professional responsibilities.

## Discussion

In the earlier years after the national roll-out of HPs, two doctors working and living in Malawi noted on the importance of HPs, saying that *“the health passport is treated with reverence*” by patients and that *“the booklet provides a complete and integrated record of immunisations*,* preventive health care priorities*,* major medical morbidities and a continuation record of clinical encounters”* [39]. They expressed enthusiasm that patient-held records empowered patients in managing their own healthcare and promoted transparency in the practitioner-patient relationship and strengthened trust.

Our study shows that the current use of the general HPs is hindered by low patient literacy and language challenges. We acknowledge that our results are drawn from the experience and practice of a small sample of healthcare professionals from facilities in Zomba district, and the situation at national level may reveal additional dimensions to these findings. Our study fills an important silence in the literature on the use and effectiveness of general HPs in Malawi and may provide a foundation for a larger study.

We found that most facilities in Zomba District, particularly rural government clinics, did not keep files for out-patients on their premises, a situation that was also described by Tough in the districts of Chikwawa and Blantyre in 2018 [17]. HPs constituted the only medical record for the majority of the population. While the HPs maintained their intended place in the workflow of patient visits to health facilities, the main problems were with their actual use by professionals: poor record-keeping, illegible handwriting, inconsistent use of shorthand and abbreviations on the part of professionals. Patients valued HPs as a means of accessing care rather than a source of information given that they rarely understood what was written. The loss of value in HPs is significant because it is an indicator of the trust that patients have in the treatment and care they receive and of the quality of the information that is communicated by and to the patients during consultations. A study in the Northern region of Malawi, found that over 82% of patients were dissatisfied with the provider communication, finding that professionals failed to explain the reason for medical tests and ignored what patients told them during consultations [40]. The *Malawi Charter of Rights for Patients*, formulated by the Medical Council of Malawi, outlines key patient rights, including access to quality information and health education, and participation in care [41]. HPs are important for facilitating these rights hence improving their use should be a priority.

Participants in the focus group discussions noted that their perception or awareness of gaps in the recorded clinical visits within patient HPs influenced the extent to which they consulted past medical history. As a result, HPs were seen as less effective in supporting healthcare professionals in making faster or more accurate diagnoses. Most practitioners reported relying primarily on their own observations during consultations, as well as on the patient’s or guardian’s account, rather than on the information recorded in the HPs.

The incompleteness of HP notes, resulting from practitioners either failing to record required information or leaving sections unfinished, was particularly noted in the case of maternal and child HPs [3, 12, 16]. Efforts have been made to improve the design of these HPs so that they collect better data. The women HP was supplemented with separate cards for family planning data [16]. The child health booklet was revisited in 2023 to improve the recording of head circumference measurements necessary for the early detection of hydrocephalus [4]. Another study found that woman HPs lacked clear instructions for healthcare providers on standard care and condition management and were of limited informational value to pregnant women [3].

Less attention has been given to the use and effectiveness of the general HP, an area where our study provides insights. Our study gave participants an opportunity to reflect on the role that HPs have within the overall health system and on the implications that ineffective use of HPs has on the quality of care delivered and on the wider information needs of the patient. In addition to the uses of HPs listed in Table 1, we found that HPs were important to the process of the referral of patients for further care at larger hospitals, and for deriving cause of death alongside other records. An important point was noted with regard to the use of stamps and signature as a mark of official records, which is important for referrals.

The role of HPs as an educative and informative tool for patients was found to be uncertain because patients struggled to understand what was written in their notes. Low patient literacy was an important factor, but our study suggests that a lack of shared medical vocabulary in local languages between patients and professionals is a major factor for the quality of information communicated. This aspect has been overlooked in previous studies.

Other studies have found large gaps in patient knowledge about malaria, or maternal and child health, all being areas in which large efforts and funding had been allocated over the years [14].

Other studies showed that cases of malaria with anaemia and diarrhea with severe dehydration go undiagnosed and develop into severe cases [42]. While this has been attributed to practitioners’ insufficient knowledge of case definitions, our research also raises the possibility that language barriers may contribute to the misdiagnosis of conditions such as malaria due to challenges in patients expressing their symptoms in English or clinicians being able to ask about symptoms and conditions in the language of the patient.

Establishing a shared vocabulary to express symptoms and conditions in local languages has the potential to improve the quality of the information that is exchanged during consultations. Patients participating in a recent study on access to health care perceived health care providers to exercise power through their communication practices, mis-behaviour, and language used to explain health problems [43]. This supports our recommendation that vocabulary training in vernacular should complement training in communication and empathy skills for professionals.

High workload and understaffing also act as additional barriers that affect the quality of the records. However, improving the availability of staff is not sufficient on its own, and regular training in record keeping, data use and analysis is needed [20]. Health practitioners, regardless of whether they work in rural and under resources clinics, should be given good access to information and to comprehensive training that includes the correct use of medical terms, best practices for patient identification, and guidelines for using abbreviations and signatures in medical records.

The transition towards eHealth and digital data collection may partly explain the absence of recent legislation or guidelines explicitly addressing the use of handwritten patient records (HPs). Notably, only the National Health Information System [1] includes a dedicated section on the use of HPs, a focus that is absent in the subsequent National Health Policy [44]. Unlike countries such as South Africa and Zimbabwe, which have established formal guidelines for documentation in patient records, no such protocols currently exist in Malawi [45, 46]. This is despite the fact that HPs continue to be used for data verification of national statistics by checking and reconciling figures between HPs, registers and electronic systems [11, 14].

The use of health passports is not limited to the activity of data recording but carries legal and ethical implications. These are: records ownership, access to information, the use of medical records in court, and proper documentation of informed consent. In recent years, the right of patients to access their health records has come under focus in many countries [47]. In Malawi, such patient rights, especially for the poor and those in rural areas, are to some extent facilitated by the use of HPs and thus improving their effectiveness should be seen as a priority.

### Recommendations

Encouraging health professionals to use HPs more efficiently and educating patients on the importance of HPs may result in a turning around of the current situation.

The recommendations emerging from this study can be summarised as follows:

1. There is a need to improve record keeping by health professionals. This could be achieved in the short-term by formulating clear and appropriate guidelines at facility or district level, and through better training and/or supervision on the job.
2. Communication with patients can be strengthened by taking steps to improve health literacy and building trustful patient-provider interactions.
3. The development of a shared vocabulary for medical terms in local languages can be a step toward restoring trust in the quality and quantity of the information that can be communicated during consultations.
4. Current health policies should give necessary attention to issues of record keeping and thus encourage both patients and professionals to value medical records.

Addressing most of these recommendations require a long-term approach involving many stakeholders. For example, there may be a case for reviewing curricula for medical/health degrees to incorporate specific content or training on record keeping and language for health.

The development of a shared medical vocabulary in local languages does fill an existing gap, but will require adequate investment, expertise and a sensible integration as a useful resource for practitioners and patients. The current situation of language use in health is unsatisfactory.

It is well known that adoption of any health systems depends on whether they can support various tasks, and work-systems practices. HPs are perceived to be consistent with the current work practices in most facilities especially in rural areas. Therefore, guidelines must acknowledge the role of the HPs and seek to address areas of concerns, some of which being highlighted in this study.

Removing barriers such as costs is also important for ensuring equitable access to health [48]. Attention should be given to addressing inequalities that may have been introduced unintentionally by programs that prioritise one segment of the population over another. This has led to some patients placing more value on HPs than others, for example women and under fives, rather than men or the youth.

### Limitations

Although this research improves our understanding of the use and effectiveness of general HPs in Malawi, it does not cover all the relevant dimensions in depth. The conclusions of this study are drawn from a very small cohort and from a specific area. Hence some bias in our results and interpretation is possible. The issues we tackled were complex and linked to areas beyond health such as education (literacy), language resources but also culture and societal norms. These deserve to be looked at in more depth in order to inform effective solutions. Improving the perception of value towards HPs and any other health records in general requires a strategic and pragmatic long-term approach.

## Conclusion

This study contributes to a better understanding of the use and effectiveness of general HPs in Malawi. The findings suggest that HPs remain vital records in the healthcare system in Malawi but that poor practitioner use, language barriers and patient literacy weaken their effectiveness as a tool that supports care. To restore their intended effectiveness, there is an urgent need to improve record-keeping practices among healthcare professionals. In doing so, it may also help to re-establish the value that professionals place on the use of HPs, reinforcing their role as vital tools in patient care and continuity. There is also a need to educate patients on the value of HPs. The effectiveness of HPs depends on verbal explanations that professionals provide to patients regarding the recorded notes. The information exchanged, however, is minimal and few practitioners explain symptoms and diagnosis to patients. Professionals believe that language, educational and terminology gaps are too wide for them to be optimistic about what depth of information can be transmitted during consultations. Therefore, there is a need to improve the overall literacy of patients and to achieve a shared medical vocabulary in local languages. The use of language resources, such as medical dictionaries or interpretation services, may facilitate improved understanding and communication.

## Supplementary Information

Supplementary Material 1.

## Figures and Tables

**Fig. 5:**
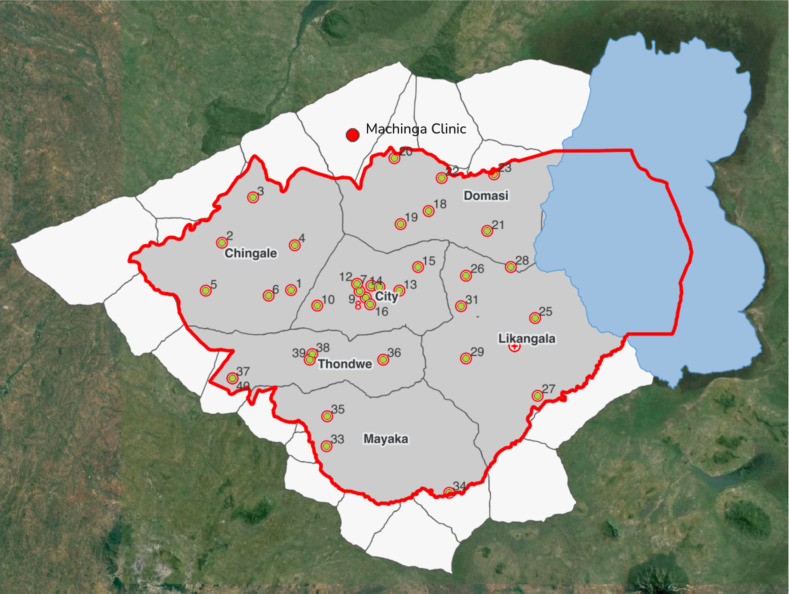
Study area showing all clinics under the supervision of the District Health Office. Participants were based at the following clinics. In Chingale area: Mmambo (location number 3 on the map), Chipini (location 17), in the City area: Matawale (location 7), Chanco (location 8), Naisi (location 10), in the Domasi area: H. Paker (location 14), Bimbi (location 16), Machinjiri (location 32). In white we show the catchment areas of these clinics. Machinga clinic is outside of Zomba district but serves population in a shared catchment area

## Data Availability

Data is provided within the manuscript or supplementary information files. Additional sample images from HPs can be obtained on request from the main author. Some data that can be made published is available at: https://osf.io/f6jgk/.

## Appendix 2

**Table 7:** List of abbreviations found in HPs with meaning given by health professionals

Notation	Meaning of abbreviation	Type
ABD	Short for abdominal, e.g. abdominal pains.	Anatomy
C/O	Short for “complains of”	Charting
CR	It could stand for “Clinical Response”	Charting
D	Diagnostic (instead of Dx). E.g. D septicaemia	Charting
DOA	Date of Admission	Charting
HX	History, e.g. HX of asthma = a history of asthma	Charting
O/E	On Examination, e.g. O/E stable	Charting
P	Plan (meaning Treatment Plan)	Charting
PE	Physical Examination, E.g. hoarseness of voice	Charting
PTC	Probably a mistake, or PC = Presenting Complaint	Charting
RC	Possibly means related condition	Charting
RX	Treatment	Charting
X	Diagnosis	Charting
Δ	Diagnosis; change	Charting
ARI*	Acute Respiratory Infection	Diagnosis/Condition
BH	Not known by most professionals.	Diagnosis/Condition
ERF	Possibly meaning established risk factors.	Diagnosis/Condition
G/E	Gastroenteritis	Diagnosis/Condition
LPR	Laryngopharyngeal Reflux	Diagnosis/Condition
PID	Short for pelvic inflammatory disease (PID) is an infection of the female reproductive system, which includes the womb, fallopian tubes and ovaries.	Diagnosis/Condition
RTI	Respiratory Tract Infection	Diagnosis/Condition
URTI*	For Upper Respiratory Tract Infection	Diagnosis/Condition
AMOXYL*	Amoxicillin (recommended in MTG for treatment of pneumonia in children)	Drug
ASA*	Aspirin	Drug
CTX	CTX usually stands for ceftriaxone, an antibiotic used to treat various bacterial infections. But in Malawi professionals suggested that this usually stands for co-trimoxazole commonly utilised in medical settings for the treatment of respiratory infections, urinary tract infections, and certain types of pneumonia, particularly in immunocompromised patients.	Drug
DCN	Doxycycline is an antibiotic that is commonly used to prevent malaria.	Drug
DETROLAM	DETROL LA treats the symptoms of overactive bladder (leaks, strong sudden urges to go, going too often).	Drug
DEXA	Dexamethasone	Drug
GENTA*	Gentamicin, a broad spectrum antibiotic used to treat severe bacterial infections, it is usually administered intravenously. It is recommended in MTG for treatment of pneumonia in children.	Drug
METRO	Metronidazole, an antibiotic that is used to treat a wide variety of infections.	Drug
NEO DEXA	Neomycin/dexamethasone. Neomycin is an antibiotic. It is used to treat bacterial infections. Dexamethasone is a steroid. It is used to treat the inflammation associated with bacterial infections of the eye.	Drug
PCM*	Short for “paracetamol”	Drug
SP*	Drug used for malaria (sulphadoxine-pyrimethamine (SP) Date on the HP where this was used was 2006.	Drug
FBC*	Full Blood Count, a type of blood test.	Laboratory
HCG*	Tumour markers - HCG. Can also be interpreted as pregnancy test.	Laboratory
MRDT*	Malaria Rapid Diagnostic Test.	Laboratory
NEG	Negative.	Laboratory
PITC*	HIV test, stands for Provider-Initiated HIV Testing and Counselling, E.g. PITC neg.	Laboratory
POS	Positive.	Laboratory
VDRL*	“Venereal Disease Research Laboratory (VDRL)” test is a blood test designed to assess whether you have syphilis, a sexually transmitted infection (STI). E.g. VDRL neg.	Laboratory
BD*	Twice a day, for example, DCN 100 mg bd	Pharmacy
OD	Once a Day, OD 0.2.5–4/6	Pharmacy
P/C*	p.c: after meals (Latin: post cibum). Indicates to take the medicine after a meal.	Pharmacy
TDS*	Three times a day	Pharmacy
TDT	Three times daily	Pharmacy
BF	BF = Breathing Frequency	Vital Signs
O/S	Oxygen Saturation	Vital Signs
PFT	Pulmonary Function test, E.g. BF:+PFT SEEN 6050	Vital Signs
TEMP	Temperature, e.g. TEMP: 37.8	Vital Signs
WGT	Weight, e.g. WGT:6.8 KG	Vital Signs

Abbreviations marked with asterix appear in the MSTG
