# Supplementary material for: Revisiting the use and effectiveness of patient-held records in rural Malawi
Source: BMC Health Serv Res. 2025 Jun 3;25:792. doi: 10.1186/s12913-025-12844-0 (PMC12135469; doi:10.1186/s12913-025-12844-0)
Supplement: Supplementary file 1 — Supplementary Material 1. [file 12913_2025_12844_MOESM1_ESM.pdf]

FOCUS GROUP DISCUSSION GUIDE

PART 1

Health Passports

Our research found that healthcare professionals do not often use the information in the healthcare passports (HP). Some reasons include: the information may be incomplete, and patients lose or not present their HP during consultations. We also found that patients do not understand what is written in their HP. We aim to investigate this aspect further.

1) How do healthcare professionals use the information in the health passports?

2) Have you done any training on how to record notes, or how to make entries in the health passports?

   Are there symbols that are commonly used? (symbols for tests, symptoms, conditions, signs, medications, etc.)

3) Do patients ask for clarifications about what is written on their health passports?

4) Do you discuss with patients what is written on their health passports during consultation?

5) What value do you think healthcare professionals place on HPs?

6) What value do you think patients place on HPs?

7) Do you think there is a case for writing symptoms and diagnoses in Chichewa or the language of the patient in the health passports?

8) How could health passports become more useful to healthcare professionals and patients?

The impact of (perceived) low literacy of patients

Perceived low literacy of patients was given as a source of language challenges. Doctors also were less likely to provide more details to patients about their conditions if they perceived the literacy levels to be low. We want to learn more about the importance of this factor.

1) What do you think is the impact of low literacy on the quality of information exchanged during consultations?

2) In what way do you think low literacy affect the ability of patients to express their symptoms and conditions?

3) In what way do you think literacy could or does affect the ability of patients to express to understand their diagnosis and treatment?

4) What interventions do you think could help mitigate the problems with patients' literacy?

Issues of privacy

Patients expressed that a lack of privacy prevented them from freely expressing their symptoms or underlying conditions during consultations.

1) To what extent do you think a lack of privacy affects good communication?

2) In what way do you think a lack of privacy affects the quality of healthcare?

3) What kind of privacy situations occur?

4) What can be done?

PART 2

Vocabulary

In our study, we found evidence that healthcare professionals would find useful if they had dictionaries of common phrases used to express symptoms, medical conditions, or treatment. We would like to understand whether the availability of translators or dictionaries could help improve communication.

Bilingual Medical Dictionary

1) Do you struggle to express the diagnosis or their medical conditions to patients due to language challenges?
   a. These can be due to language differences between you and the patient
   b. Or finding the right vocabulary on the spot
   c. Or other

2) Has your formal medical training given you the necessary preparation to help you handle different issues that may arise in patient communication?

3) If provided, how useful would a medical dictionary with Chichewa expressions for symptoms, disease or body organs be?

4) Do you think a provision for interpreter services in health clinics is needed?

   a. What challenges do you think there will be?
   b. Would having an interpreter available lower the usefulness of a dictionary?

List symptoms/questions asked in the diagnosis of the following

Diagnosing Malaria for Children

English

**ENGLISH**	CHICHEWA

Diagnosing Malaria for adults

ENGLISH	**CHICHEWA**

Diagnosing diarrhoea conditions

<u>ENGLISH</u>	**<u>CHICHEWA</u>**
